# Proceedings of the 2nd BEAT-PCD Conference and 3rd PCD training School: part 2: Valencia, Spain. 18-21 April 2017

**DOI:** 10.1186/s12919-018-0099-8

**Published:** 2018-03-05

## P01 Development of a bioinformatics analysis method for diagnosis of Primary Ciliary Dyskinesia

### Erif Alik Server

#### Acibadem Üniversitesi, İstanbul, Turkey

Primary ciliary dyskinesia is a rare, autosomal recessive genetic based disease causing functional and/or structural defects of cilia. However, there are little information about the genetic basis of this disease. By this time, we know that, mutations on the DNAI1 and DNA5H genes are responsible for encoding of dynein proteins of cilia, causing this disorder. Discrimination of the PCD from other diseases like Cystic fibrosis is quite problematic. Discrimination and treatment will be easier if the genetic basis of the PCD is known. In the proposed work, we plan to carry out whole genome exome sequencing on 3 families with PCD siblings. Each these families have healthy parents and two sick children. As a result of exome sequencing, we will able to identify common SNP’s and/or affected common genes related with PCD. Then, the data obtained from exome sequencing will be used as the input to PANOGA to identify affected pathways. Thus, we will be able to study PCD aetiology via affected pathways. Eventually, we aim to provide a novel marker for diagnosis of PCD.

## P02 Quality of life in patients with primary ciliary dyskinesia: a systematic review

### Laura Behan^1,2^, Bruna Rubbo^1^, Jane S Lucas^1^, Audrey Dunn Galvin^2^

#### ^1^NIHR Southampton Biomedical Research Centre, University of Southampton and University Hospital Southampton NHS Foundation Trust, Southampton, UK; ^2^School of Applied Psychology, University College Cork, Cork, Ireland

**Background:** As in most rare diseases, little is known on how primary ciliary dyskinesia (PCD) affects the patient’s daily functioning and mental wellbeing. We aimed to identify all studies that examined the psychosocial impact that PCD has on the lives of patients. **Methods:** We searched MEDLINE, EBSCO, Cumulative Index to Nursing and Allied Health Literature (CINAHL), PsycINFO and EMBASE for qualitative and quantitative studies. **Results:** We found 260 studies, of which 14 were included. Five of the included studies had qualitative approaches. Increasing age was associated with worsening of respiratory symptoms, physical, and mental domains of health-related quality of life, with a greater decline compared with reference populations. Patients felt physically impaired, e.g. less energy and difficulty keeping up with others. Social implications included embarrassment and a sense of isolation, with patients concealing symptoms and/or their diagnosis. Isolation intensified with lack of public and professional awareness of PCD. Patients were emotionally affected by PCD and expressed anxiety about getting sick or when thinking about their future health. Treatment burden was an issue for patients, whilst acknowledging treatments improved symptom. **Conclusion**: Health-related quality of life decreases with age in patients with PCD. For all age groups, PCD adversely affects physical, emotional, social functioning, and treatment burden. Interventions that ameliorate these issues need investigation.

## P03 Estimation of cell number in a three-dimensional cell cluster comparison of different 3D nuclei calculating methods

### Marek Kraft^1#^, Zuzanna Bukowy-Bieryllo^2#^, Agnieszka Fedoruk-Wyszomirska^3^, Maciej Dabrowski^2^, Eliza Wyszko^3^, Magdalena Pikulska^2^, Michal Witt^2^, Ewa Zietkiewicz^2^

#### ^1^Poznan Technical University, Poznan, Poland; ^2^Institute of Human Genetics, Polish Academy of Sciences, Poznan, Poland; ^3^Institute of Bioorganic Chemistry, Polish Academy of Sciences, Poznan, Poland

##### **Correspondence:** Zuzanna Bukowy-Bieryllo (zuzanna.bukowy-bieryllo@igcz.poznan.pl); Agnieszka Fedoruk-Wyszomirska

^#^equal author contribution.

Efficient counting of cell numbers in three-dimensional structures (cell clusters, spheres or aggregates) often poses a challenge for the existing image analysis software. We present a novel approach to the problem, for the use on fluorescence-stained three-dimensional volumes (z-stacks) obtained using confocal microscope. The method combines the 2D segmentation of nuclei slices using grabcut algorithm, followed by three-dimensional region labelling and nuclei assembly. The software has been developed with the use of Python software, as well as the Bio-Formats, Open-CV and SciPy libraries. Time consumption, precision and scalability of the method have been compared with other available nuclei calculating methods (manual counting and available ImageJ plugins). We also explored the possibility to use of our algorithm on z-stacks obtained using regular fluorescent microscope.

This research has received funding from the National Science grant number 2013/09/D/NZ4/01692.

## P05 *In vitro* differentiation of respiratory epithelial cells in the sequential culture model

### Maciej Dabrowski^1^, Zuzanna Bukowy-Bieryllo^1^, Agnieszka Wyszomirska-Fedoruk^2^, Magdalena Pikulska^1^, Michal Witt^1^, Ewa Zietkiewicz^1^

#### ^1^Institute of Human Genetics, Polish Academy of Sciences, Poznan, Poland; ^2^Institute of Bioorganic Chemistry, Polish Academy of Sciences, Poznan, Poland

##### **Correspondence:** Maciej Dabrowski (maciej.dabrowski@igcz.poznan.pl)

*In vitro* culturing of the respiratory epithelial cells is a primary step in studies on the structure, functioning and differentiation of the respiratory epithelium (RE). It is also used during diagnostic process of primary ciliary dyskinesia, to obtain cilia without secondary changes caused by infection load. Two most commonly used methods of culturing airway epithelial cells are: the air-liquid interface (ALI) culture and the sequential culture (Jorissen’s method). In the sequential culture cells are first cultured adhered to a layer of collagen gel, after reaching confluence, the cell sheets are released from the collagen layer and transferred into suspension. Although much is known about the expression of genes during differentiation of airway epithelial cells cultured at the ALI culture, not much information exists about the epithelium differentiation chronology in the sequential culture of RE cells. Here, using a panel of antibodies against proteins characteristic for different epithelial cell types, we characterized the different stages of epithelial differentiation. Basal cells of the epithelium were monitored using antibody against p63 protein, antibody against MUC5AC protein was used to detect secretory (goblet) cells and antibody against CC10 was used to detect club cells (previously known as Clara cells). Ciliated airway cells were detected using antibodies against cilia-specific proteins - acetylated α-tubulin or RSPH4A. Expression of proteins was analyzed at different time-points of the culture (at culture start, after proliferation period, at the beginning and the end of cell differentiation). In addition to the protein expression, the time-dependent changes in the gene expression level were monitored, using multiplex RT-PCR with primers against cDNA of several genes: *DNAH5, CCDC40, ZMYND10, RSPH4A, CFTR, GAPDH*.

Study supported by the National Science Centre grants 2013/09/D/NZ4/01692 ZBB, 2014/13/B/NZ2/03858 EZ, 2016/20/T/NZ4/00525 MD.

## P06 Preliminary results of whole exome sequencing in Turkish primary ciliary dyskinesia patients - Hacettepe University Experience: “Three candidate genes, five novel and two known mutations”

### Nagehan Emiralioğlu^1^, Ekim Taşkıran^2^, Can Koşukçu^2^, Gökçen Dilşa Tuğcu^1^, Sanem Eşref^1^, Mina Gharibzadeh Hızal^1^, Ebru Yalçın^1^, Deniz Doğru Ersöz^1^, Nural Kiper^1^, Mehmet Alikaşifoğlu^2^, Uğur Özçelik^1^

#### ^1^Hacettepe University Faculty of Medicine, Department of Paediatric Pulmonology, Ankara, Turkey; ^2^Hacettepe University Faculty of Medicine, Department of Medical Genetic, Ankara, Turkey

**Background:** Primary ciliary dyskinesia (PCD) is characterized by chronic respiratory tract infections, abnormally positioned internal organs and infertility. It usually shows an autosomal recessive inheritance pattern. However consistent with its clinical variability, PCD is a genetically heterogeneous trait and nearly 32 genes associated with this disease thus far. The majority of mutations are INDELs and loss-of-function variants, despite missense mutations have been shown in a few cases. Recent technologies can detect approximately 60% of biallelic PCD mutations. However, whole-exome sequencing (WES) provides the opportunity for the (Genetically) undiagnosed primary ciliary dyskinesia patients and describe both candidate genes and novel mutations. Here we aimed to reveal the underlying genetic risk variants of PCD patients with whole-exome sequencing. **Methods:** Whole-exome sequencing (WES) was performed in 10 individuals with a history of primary ciliary dyskinesia (PCD) and who had a sibling with the diagnosis of PCD followed at Hacettepe University Faculty of Medicine Department of Paediatric Pulmonology. Causative mutations in consanguineous cases identified the underlying genetic risk variants. Allele frequency and in-house database information with homozygosity approach was used for variant filtering. Lung Diseases NGS Panel which contains 180 known and candidate genes was also utilised. Whole exome sequences and data analysis were carried out as a part of Hacettepe Exome Project, 2015-2017 (funded by Hacettepe University, TAY 2015-7335). **Results:** Bioinformatic analysis ended up with 10 different homozygous variants which include INDEL, missense and nonsense alterations in both known and candidate PCD genes. Mutation analysis showed all affected siblings are homozygous for indicated variants. Variants identified by whole exome sequencing were also confirmed and the inheritance in families showed by Sanger sequencing. We detected novel and previously reported mutations in *CCDC40*, *CCNO*, *DNAH5*, *DNAH9* and *RSPH4A* which are related to PCD. Our findings also suggest three candidate genes that were not reported in PCD patients so far. **Conclusion:** Diagnosis of patients with the detection of both diseases associated and novel mutations is an important approach for this project. Besides, with the growing next generation sequencing technologies, genetic analysis become crucial to assess the carrier family members and provide tools for prenatal diagnosis. Also, these studies could lead to develop genetic diagnostic kits or expand the existing ones for patients with PCD. The obtained data will contribute to genetic features of primary ciliary dyskinesia and will be important to show genotype-phenotype correlations. These findings will also guide to population based genetic studies of Turkish PCD patients in future.

## P07 Antibiotic resistance of *Haemophilus influenzae* in primary ciliary dyskinesia patients

### Kısmet Çıkı^1^, Selay Demirci^2^, Banu Sancak^2^, Nagehan Emiralioğlu^3^, Gökçen Dilşa Tuğcu^3^, Sanem Eşref^3^, Mina Gharipzadeh Hızal^3^, Ebru Güneş Yalçın^3^, Deniz Doğru Ersöz^3^, Nural Kiper^3^, Burçin Şener^2^, Uğur Özçelik^3^

#### ^1^Hacettepe University Faculty of Medicine, Department of Paediatrics, Ankara, Turkey; ^2^Hacettepe University Faculty of Medicine, Department of Clinical Microbiology, Ankara, Turkey; ^3^Hacettepe University Faculty of Medicine, Department of Paediatric Pulmonology, Ankara, Turkey

**Background:** Primer ciliary dyskinesia (PCD) is a rare, genetic disease characterized by cilia dysfunction. Recurrent lower respiratory tract infection and bronchiectasis play an important role in morbidity. The most frequently isolated microorganisms in the respiratory tract are *Haemophilus influenzae*, *Streptococcus pneumonia*, *Staphylococcus aureus*, *Pseudomonas aeruginosa* and non-tuberculosis mycobacteria. Frequent respiratory tract infections often lead to the use of antibiotics in patients. **Aim:** Our aim in this study is to investigate the relationship between antibiotic use and resistance in PCD patients with *H. influenzae* in respiratory tract specimens during the last year. **Methods:** The sputum and bronchoalveolar lavage cultures of patients diagnosed with PCD in the Department of Paediatric Chest Diseases of Hacettepe University were examined for antibiotic susceptibility of *H. influenzae* in 2016. Antibiotic susceptibilities of isolates were determined by disk diffusion method. Antibiotic use of these cases was evaluated retrospectively in the last year. **Results:** *H. influenzae* were detected in 74 cases (33 M/ 41 F and the age range is 3-25 years and the mean age is 13,9 years.) of 120 PCD patients which were seen in 2016. 5 of *H. influenzae* cases were detected in the bronchoalveolar lavage and 69 in the sputum samples. 11 patients had another microorganism concurrent with *H. influenzae*. In 4 of the cases synchronous *S. pneumoniae* was detected. Antibiotic susceptibilities in cultures were shown, the highest resistance was detected against trimetoprim sulfamethoxazole (24,1%), ceftazidime (11,1%), ampicillin (8,2%) and amoxicillin-clavulanate (6,5%). When patients were evaluated for antibiotic use within the past year, 48 patients were treated with amoxicillin clavulanate, 28 patients with macrolide group and 12 patients with TMP-SXT. Resistance was detected in 58.3% of patients using TMP-SXT. Only the use of TMP-SXT showed a statistically significant relationship with the development of antibiotic resistance (p = 0.005). **Conclusion:** TMP-SXT is one of the most rapidly developing antimicrobial resistance clinically, despite its low use rate. As a result of this study, TMP-SXT resistance was detected in 58.3% of the patients using TMP-SXT in the last year. The low resistance to amoxicillin-clavulanate supports the use of beta-lactam antibiotics with beta-lactamase inhibitor in *H. influenzae* infections in PCD cases.

## P010 The effect of L-Arginine on Cilia Beat Frequency in PCD patients, non-PCD referrals and healthy controls

### Panayiotis Kouis^1^, Andreas Hadjisavvas^2,3^, Nicos Middleton^4^, Stefania Papatheodorou^1^, Kyriacos Kyriacou^2,3^, Panayiotis K. Yiallouros^5^

#### ^1^Cyprus International Institute for Environmental & Public Health, Cyprus University of Technology, Limassol, Cyprus; ^2^Department of Electron Microscopy and Molecular Pathology, Cyprus Institute of Neurology and Genetics, Nicosia, Cyprus; ^3^Cyprus School of Molecular Medicine, Cyprus Institute of Neurology and Genetics, Nicosia, Cyprus; ^4^Department of Nursing, Cyprus University of Technology, Limassol, Cyprus; ^5^Medical School, University of Cyprus, Nicosia, Cyprus

**Background**: Nasal nitric oxide (NO) is low in Primary Ciliary Dyskinesia (PCD) and has been found to correlate with compromised Ciliary Beat Frequency (CBF). Few studies have examined the potentially therapeutic effect of increasing the production of endogenous NO in PCD. In this study we assessed the effect of increasing substrate of NO synthases, L-Arginine, on CBF in biopsies of human respiratory ciliated epithelium. **Methods**: A total of 28 suspect cases referred for PCD diagnostic testing and 8 healthy controls underwent nasal brushing. Obtained epithelial cells were divided between three culture medium 199 solutions, containing different levels of L-Arginine (0.33mM as baseline, 1mM and 10mM). CBF measurements were obtained at 37°C and 25°C at 1, 3 and 24 hours after sample acquisition. **Results:** PCD diagnosis was confirmed in 8 subjects and excluded in 20. Among PCD subjects, ciliary motility was characterized by rotational (n=5) or dyskinetic (n=3) beating. At 37°C, compared to baseline, higher levels of L-Arginine resulted in up to 9% CBF increase at 1 hour (p=0.007), up to 9% CBF increase at 3 hours (p<0.001) and up to 12% CBF increase at 24 hours (p=0.002). Similar although smaller scale increases were recorded at 25°C. The effect of L-Arginine was time dependent (interaction p=0.002) and was similar in PCD patients, non-PCD referrals and healthy controls (interaction p=0.800). **Conclusions:** L-Arginine increases CBF and merits to be evaluated as a potential stimulator of mucociliary clearance in ciliary disorders with residual motility. Larger human studies are needed to confirm these findings.

## P011 The role of Rabconnectin3a in cilia length regulation

### Bárbara Tavares, Sara Pestana, Susana S. Lopes

#### NOVA Medical School Faculdade de Ciências Médicas, Chronic Diseases Research Centre, CEDOC, Universidade Nova de Lisboa, Campo Mártires da Pátria, 130, 1169-056 Lisboa, Portugal

Using the zebrafish mutant for the *deltaD* gene (*dld*^*-/-*^), we showed that Notch signaling was involved in the control of cilia length in the cells of the fish laterality organ, the Kupffer’s Vesicle (KV). A microarray analysis of the list of genes differentially expressed in KV cells from dld^-/-^ mutants and wild-type embryos, identified a gene 16 fold down-regulated. This gene – *rabconnectin3a* or *rbc3a* codes for a large, ~325 kDa protein, highly conserved in multicellular organisms, which associates with its obligate binding partner, Rbc3b and subunits of the v-ATPase complex. Rbc3a was implicated in the regulation of the activity of the V-ATPase. Its absence has been shown to decrease the acidification of intracellular vesicles, which can affect not only the cellular vesicular trafficking, but also the cytosolic Ca2+ concentration. This gene has been associated with Notch signalling in Drosophila and mammalian cells through the regulation of the V-ATPase activity. Rbc3a has also been associated with vesicular acidification in zebrafish hair cells and with vesicular endocytosis and maturation in zebrafish neural crest migration. Furthermore, it was found that V-ATPase H+ pump, together with SNX10, regulated ciliogenesis both in vitro and in vivo. Using a Morpholino against *rbca3* (Rbc3a MO), we caused a decrease in the cilia length of the KV and consequent randomization of organ *situs.* We propose that the decrease in ciliary length of *dld*^*-/-*^ mutant KV cells is partially caused by the deregulation of the V-ATPase pump. Using the vital dye LysoTracker Red we showed the decrease of acidic vesicles both in rbc3a morphant and *dld*^*-/-*^ mutant embryos. We co-localized the V-ATPase to the apical membrane and to the basal body and used concanamycin, a specific V-ATPase inhibitor, to assess its effect on cilia length and we registered a similar expected reduction. We show that the reduction in *rbc3a* transcription levels negatively regulates the V-ATPase activity, resulting in a generalized decrease of the late endocytic vesicular trafficking, and in a shorter ciliary length.

## P012 Late diagnosis of the PCD is still a great risk

### Vendula Martinu, Petra Dvorakova, Jiri Uhlik, Petr Pohunek

#### Paediatric Pulmonology, Paediatric Department, 2^nd^ Faculty of Medicine and University Hospital Motol, Prague, Czech Republic

We are presenting a typical case of a PCD patient with late diagnosis. Nine-year-old girl with dextrocardia and polysplenia and with recurrent respiratory infects was referred to our department for a suspicion of a PCD. She was born full-term with short ICU admission and she has had rhinorhea since birth. She has got a chronic wet cough all year round. During physical activity, she has suffered from dyspnoea and worsening of cough. She has complained of hearing problems and persistent ear secretions. She had pneumonia twice before the age of eight. The PICADAR score of our patient was 12. HRCT examination revealed chronic infiltrations, collapse of the right lower lobe, bronchiectasia and mild hilar and mediastinal lymphadenopathy. Lung function examination at the time of diagnosis detected FVC 1.48 L (77% predicted) and FEV1 0.95 L (58% predicted) with only mild response to bronchodilator. Chronic bronchitis with purulent mucus production and tracheomalacia was found during flexible bronchoscopy. Cultures from bronchoalveolar lavage revealed *Haemophillus influenzae.* ENT examination found a mild conductive hearing disorder and chronic secretory otitis. Impressive history suggested PCD. CF was ruled out; laboratory findings did not show any immunopathology. Nasal NO was 10.2 nl/min, HSVA demonstrated akinetic cilia. Ultrastructural examination showed absent outer dynein arms (ODA) in ciliary axonema, inner dynein arms were present. Recently introduced immunofluorescence examination revealed DNAH5 deficit, which supported our diagnosis of PCD with ODA defect. We have initiated the complex therapy consisting of targeted antibiotic therapy, inhaled mucolytic therapy, inhalation of ICS/LABA and chest physiotherapy including flutter. Thanks to this intensive therapy the patient’s condition has significantly improved despite the delayed diagnosis. This case indicates that despite typical symptoms the diagnosis of PCD may still be unnecessarily delayed. Increased awareness of the disease among both medical professionals and lay public is highly desirable.

## P014 Ten years’ experience of primary ciliary dyskinesia diagnostic testing

### Bruna Rubbo,^1^ Laura Behan,^1^ Ana Paula Lima de Queiroz,^2^ Patricia Goggin,^1^ Claire Jackson,^1^ Samantha Packham,^1^ Woolf Walker,^1^ Jane Lucas^1^

#### ^1^Primary Ciliary Dyskinesia Centre, NIHR Biomedical Research Centre, Southampton, UK; ^2^ Bahiana School of Medicine and Public Health, Salvador, Brazil

**Background:** The Southampton primary ciliary dyskinesia (PCD) Diagnostic Centre has been commissioned by NHS England since 2006. The centre contains state of the art diagnostic facilities and specialised multidisciplinary team dedicated to PCD diagnosis and management. We aimed to describe the characteristics of patients diagnosed at the centre. **Methods:** We conducted a cross-sectional study using data from local PCD registry, electronic health records and medical notes for all patients diagnosed with PCD in Southampton from 2006 to 2016. **Results:** Of 1,720 referrals, we diagnosed 142 patients with PCD (8%). Diagnosis was always based on at least one diagnostic test. The number of tests has increased in the last 5 years particularly in genotyping (65% *vs* 56% before 2011). Nasal nitric oxide was measured in 82% of patients over 5 years of age. Testing was done on samples collected from patients attending clinic (77%) and on samples sent by courier (23%). Overall, 84% patients had high-speed video analysis, of which 70% showed predominantly static cilia and only 1.7% were deemed normal, 97% had transmission electron microscopy, of which 70% showed ‘hallmark’ defects, and 59% were genotyped. Median age at initial assessment was 10 years (IQR 3 to 23.5 years), younger for the 43% with situs abnormalities (7 *vs* 12 years, p=0.062). 11% had congenital heart disease, considerably higher than reported elsewhere (3-6%). 72% experienced neonatal respiratory symptoms, of which 58% needed respiratory support. Mean FEV_1_% was low in both children and adults (79% *vs* 71% respectively, p=0.26) at the time of diagnosis. **Conclusion:** We found a high prevalence of congenital heart disease and neonatal respiratory symptoms. Lung function was impaired in both children and adults. These findings highlight the need for early diagnosis and care for PCD in specialised centres.

